# Glucagon-like peptide-1 receptor agonists as add-on therapy to insulin for type 1 diabetes mellitus

**DOI:** 10.3389/fphar.2023.975880

**Published:** 2023-03-16

**Authors:** Xinrui Tan, Xiongfeng Pan, Xiaochuan Wu, Songjia Zheng, Yuyao Chen, Donghai Liu, Xingxing Zhang

**Affiliations:** ^1^ Department of Pediatrics, The Second Xiangya Hospital, Central South University, Changsha, Hunan, China; ^2^ Department of Epidemiology and Health Statistics, Xiangya School of Public Health, Central South University, Changsha, Hunan, China

**Keywords:** glucagon-like peptide-1 receptor agonists, meta-analysis, type 1 diabetes, adult, adjunctive therapy

## Abstract

**Background:** To assess the efficacy and safety of glucagon-like peptide-1 receptor agonists (GLP-1 RAs) used as an adjunct to insulin therapy in adults with type 1 diabetes.

**Methods:** A search of electronic databases (Medline, Embase, and the Cochrane Central Register of Controlled Trials) from 1 January 1950 to 23 May 2021 was conducted to find randomized controlled trials. The primary outcome was the change in HbA1c. Eight efficacy and six safety secondary endpoints were evaluated *via* meta-analysis. Weighted mean difference (WMD) and odds ratio (OR), alongside 95% confidence interval (CI), were calculated using the random effects model.

**Results:** Among 1,379 candidate studies, 11 trials comprising 2,856 participants satisfied the inclusion criteria. Overall, GLP-1 RA adjunctive therapy reduced HbA1c by −0.21% (95% CI, −0.33 to −0.10), weight by −4.04 kg (−4.8 to −3.27), systolic pressure by −2.57 mmHg (−4.11 to −1.03), and diastolic blood pressure by −1.02 mmHg (−1.99 to −0.06). In addition, there was a decrease in prandial insulin dose (WMD, −4.23 IU; 95% CI, −5.26 to −3.20), basal insulin dose (−2.40 IU; −3.93 to −0.87), and total insulin dose (−5.73 IU; −10.61 to −0.86). Moreover, GLP-1 RAs did not increase the incidence of severe hypoglycemia, diabetic ketoacidosis, or severe adverse events. However, GLP-1 RAs increased the incidence of gastrointestinal adverse events (OR, 2.96; 95% CI, 2.33–3.77).

**Conclusion:** Our meta-analysis of randomized clinical trials suggests moderate beneficial effects of GLP-1 RAs on the metabolic profile in patients with type 1 diabetes, without an increased risk of serious adverse events.

**Clinical Trial Registration:** https://www.crd.york.ac.uk/PROSPERO; Identifier: CRD 42020199840.

## Introduction

Type 1 diabetes is characterized by absolute insulin deficiency due to autoimmune destruction. Despite advances in treatments and monitoring methods, less than 20% of patients with type 1 diabetes achieve their glycemic goal (Foster et al., 2019). On the contrary, side effects such as weight gain and hypoglycemic episodes also affect patients’ compliance and confidence in insulin therapy (American Diabetes, 2021). There is much interest in the potential role of non-insulin therapies for type 1 diabetes. Glucagon-like peptide-1 receptor agonists (GLP-1 RAs) as adjuncts to insulin treatment seem to provide a plausible approach to overcome these challenges.

Theoretically, GLP-1 RA and insulin combinational treatment has a good rationale for patients with type 1 diabetes. First, GLP-1 RAs have glucose-lowering effects independent of insulinotropic properties, such as the suppression of glucagon release and the delay of gastric emptying (Andersen et al., 2018). Second, GLP-1 RAs induce satiety by activating the GLP-1 receptor in the brain, leading to bodyweight reduction, which may offset the weight gain associated with insulin therapy (Sandoval and D'Alessio, 2015). Third, due to the wide distribution of GLP-1 receptors, GLP-1 RAs have additional cardioprotective and renal-protective effects, besides its implication on established risk factors (Kristensen et al., 2019). Recently, several clinical trials have been conducted to assess GLP-1 RA and insulin combinational treatment in patients with type 1 diabetes; however, the conclusions of these studies have not been consistent.

Since an individual study may not be sufficient to provide objective evidence, and because GLP-1 RAs differ in structure, size, and pharmacokinetics, including liraglutide, exenatide daily, exenatide extended release (Exenatide ER), and albiglutide, this study carried out an up-to-date meta-analysis to assess this combination therapy in the management of type 1 diabetes. The structures, sizes, and pharmacokinetics of all of the agents are available in Supplementary Material S1.

## Methods

### Search strategy and selection criteria

Before data extraction, a predetermined detailed analysis protocol was registered in PROSPERO database, registration number CRD 42020199840 (https://www.crd.york.ac.uk/PROSPERO). The predetermined detailed analysis protocol is available in Supplementary Material S2. This meta-analysis was conducted according to the Preferred Reporting Items for Systematic Reviews and Meta-Analyses (PRISMA) guidelines. Experienced librarians designed and adjusted a broad but highly structured MeSH (Medical Subject Headings) terms search strategy. The detailed search strategy for randomized controlled trials that compared GLP-1 RA therapy and placebo in patients with type 1 diabetes is available in Supplementary Material S3. Following PRISMA guidelines, two independent reviewers (Xinrui Tan and Xiongfeng Pan) systematically searched Medline, Embase, and the Cochrane Central Register of Controlled Trials, for randomized controlled trials from 1 January 1950 to 23 May 2021. Disagreements on the eligibility of the studies were settled by involving a third reviewer (Xiaochuan Wu).

### Study selection and data extraction

Studies were selected for data extraction based around the PICOS framework: Our population comprised patients with type 1 diabetes (P), the intervention/exposure was GLP-1 RA therapy (I), the comparison was placebo therapy (C), the outcome was change in type 1 diabetes-related efficacy and safety outcomes (O), and the study design included randomized controlled trials (S). Specifically, inclusion criteria were 1) prospective, randomized, and controlled clinical trials; 2) assessment of the efficacy and safety of GLP-1 RAs as an adjunct therapy in patients with type 1 diabetes; 3) at least 8 weeks of intervention. Studies were excluded if they 1) were case reports, retrospective or crossover studies, or conference papers; 2) did not report HbA1c, body weight, or total daily insulin dose at baseline and end of trial; 3) if they included special populations (e.g., individuals with gestational diabetes); or patients with type 2 diabetes. The characteristic data, including the study duration, number of follow-ups, type of GLP-1 RAs used, and outcomes of each trial, were extracted. In order to avoid overlapping populations, this study only analyzed the results with the most comprehensive information. The longest follow-up period to evaluate the main results was used when published studies reported outcomes for various follow-up periods. Two reviewers (Yuyao Chen and Songjia Zheng) independently extracted the data using a custom data extraction template. Disagreements on the eligibility of the studies were settled by involving a third reviewer (Xiaochuan Wu). Endnote (version x9.1) was used to remove duplicate data, and then EpiData (version 3.0) was used to extract data. All the data were stored in a custom Microsoft Excel data extraction template (version 2019).

### Outcomes

Outcomes of interest included efficacy variables [HbA1c (%), mean blood glucose (MBG) (mmol/L), glucose SD (mmol/L), mean amplitude of glycemic excursions (MAGE) (mmol/L), body weight (kg), diastolic blood pressure (mmHg), systolic blood pressure (mmHg), total insulin dose (IU), prandial insulin dose (IU), and basal insulin dose (IU)] and safety variables (hypoglycemia, severe hypoglycemia, diabetic ketoacidosis, gastrointestinal disorders, adverse events, severe adverse events). The primary outcomes included HbA1c, MBG, glucose SD, MAGE, hypoglycemia, and severe hypoglycemia; the secondary outcomes included body weight, diastolic blood pressure, systolic blood pressure, total insulin dose, prandial insulin dose, basal insulin dose, diabetic ketoacidosis, gastrointestinal disorders, adverse events, and severe adverse events. Meanwhile, gastrointestinal disorders included nausea, vomiting, and abdominal pain. The definitions of hypoglycemia and severe hypoglycemia were similar to those of the investigated trials and followed the American Diabetes Association criteria (Supplementary Material S4).

### Study quality assessment

Two reviewers (Donghai Liu and Xingxing Zhang) independently assessed the eligibility of each trial, extracted data, and evaluated potential bias. The risk of trial bias assessment scheme was used to assess the risk of bias and the quality of the eligible studies as recommended by the Cochrane Collaboration. Any disagreements regarding inclusion were settled by consensus involving a third reviewer (Xinrui Tan).

### Statistical analysis

This study used Stata (version 15.0) for the meta-analysis. The weighted mean difference (WMD) and pooled odds ratio (OR) with the corresponding 95% confidence interval (CI) were calculated by random effects inverse variance mode effects meta-analysis (Cohen, 1988; Higgins et al., 2003; Pan, et al., 2020a). WMD was used to evaluate the influence of GLP-1 RAs on HbA1c, MBG, glucose SD, MAGE, body weight, diastolic blood pressure, systolic blood pressure, total insulin dose, prandial insulin dose, basal insulin dose and other markers across the studies (RileyRichard et al., 2011; Michael et al., 2019). The OR was used to evaluate the influence of GLP-1 RAs on hypoglycemia, severe hypoglycemia, diabetic ketoacidosis, gastrointestinal disorders, adverse events, severe adverse events and other safety variables across the studies (Pan, et al., 2020b). Furthermore, this study calculated the inconsistency (I^2^) to quantify this statistical heterogeneity (Higgins, 2002; DerSimonian and Laird, 2015; Pan et al., 2019). Subgroup meta-analyses were conducted to explore the sources of heterogeneity with respect to the following subgroups based on the sample and study characteristics: The impact of liraglutide in obese and overweight patients *versus* normal-weight patients, in C-peptide-positive *versus* negative patients, and long-acting GLP-1 RAs *versus* short-acting GLP-1 RAs for HbA1c reduction in patients with type 1 diabetes. Lastly, publication bias was estimated visually by funnel plots, and the Egger’s test was used to evaluate the possibility of publication bias (Egger et al., 1997). A *p*-value (two-sided) of <0.05 indicates statistically significant results.

## Results

The search strategy retrieved a total of 1,379 studies, of which 86 were from PubMed, 1076 from Embase, and 217 from the Cochrane Central Register of Controlled Trials. After excluding duplicate studies, two independent reviewers (Xinrui Tan and Xiongfeng Pan) grouped relevant eligible studies based on title and abstract screening. After excluding duplicate studies, 1,262 abstracts were reviewed, of which 1,206 were excluded. Then, the full texts of the 56 articles were assessed, and it was found that 45 articles (31 with an ineligible study design, three with a short duration of follow-up, eight lacking primary endpoints, and three duplicates) were not eligible for this study and hence were excluded. Disagreements on the eligibility of the studies were settled by involving a third reviewer (Xiaochuan Wu). Finally, 11 randomized trials were included in the final meta-analysis (Supplementary Material S5). All of the trials were published between 2015 and 2021. Eight studies assessed liraglutide, one exenatide daily, one exenatide ER, and one albiglutide. In total, 2856 patients were randomized (2023 to GLP-1 RAs and 833 to placebo) in 11 studies (Table 1).

**TABLE 1:** Characteristics of the trials investigated.

Study	Treatment	Duration, w	Number of baseline	Number of follow-up	Men, %	Mean age, y	Duration of DM^a^, y	HbA1^c^, %	BMI^b^, kg/m^2^	Weight, kg
Kuhadiya et al. (2016)	Liraglutide	12	72	63	44.4	44.8	24.1	7.6	28.8	84.8
Mathieu et al. (2016)	Liraglutide	52	1389	1389	47.7	43.7	21.4	8.2	29.5	86.2
Ahren et al. (2016)	Liraglutide	26	831	831	46.0	43.2	21.1	8.1	28.9	83.9
Dejgaard et al., 2016	Liraglutide	24	100	100	65	48.0	22.5	8.7	30.1	93.7
Johansen et al. 2020	Exenatide	26	108	105	72.4	50.3	21.1	8.3	28.3	87.7
Dejgaard et al. (2021)	Liraglutide	26	44	44	31.8	46.5	20.5	8.2	29.5	86.5
Pozzilli et al. 2020	Albiglutide	52	61	61	55.7	22.5	NA^c^	7.3	22.4	66.8
Frandsen et al. 2015	Liraglutide	12	40	36	66.7	37.8	19.0	8.8	23.5	75.4
Herold et al. 2020	Exenatide er	24	79	79	31.6	36.1	19.6	7.6	29.4	83.9
Ghanim et al. 2020	Liraglutide	26	84	64	37.5	46.2	NA^c^	7.9	31.7	89.6
Brock et al. 2019	Liraglutide	26	48	39	79.5	50.5	31.5	8.2	29.0	92.5

^a^ DM, diabetes mellitus.

^b^ BMI, body mass index.

^c^ NA, not available.

The mean age of the patients was 43.4 (SD 13.6) years, and 1455 (52%) patients were female. The mean duration of type 1 diabetes was 22.6 (SD 13.0) years and the duration of the trials ranged from 12 to 52 weeks. The patients’ mean HbA1c at baseline was 8.1% (SD 1.0%), while the mean BMI was 29.2 (SD 5.3) kg/m^2^. Six studies enrolled patients using either multiple daily injections (MDIs) or insulin pump (1679 with MDIs and 755 with insulin pump), four trials exclusively enrolled patients using MDIs (302 patients), and the Lira-Pump trial exclusively enrolled patients using insulin pump (44 patients).

Supplementary Material S6 shows the assessment of the quality of the trials according to the Cochrane Handbook for Systematic Reviews of Interventions. All 11 randomized controlled trials reported adequate randomization, allocation concealment, and blinding, and none was stopped early. Three studies had a high risk of incomplete outcome data since a relatively large number of cases were lost during follow-up. One study was classified as high risk in selective reporting because they did not report the outcome of hypoglycemia events. Three studies were considered high risk of other biases because of an imbalanced baseline or inappropriate study design.

### Treatment effect

#### Glycemic efficacy

Pooled analysis showed that GLP-1 receptor agonist adjunctive therapy reduced HbA1c levels compared to the placebo (WMD, −0.21%; 95% CI, −0.33 to −0.10) in patients with type 1 diabetes. Specifically, liraglutide led to a significant reduction in HbA1c (WMD, −0.26%; −0.38 to −0.14), while exenatide, exenatide ER, and albiglutide did not show evident efficacy. This study found no publication bias in this analysis (*p* = 0.7708).

**FIGURE 1:**
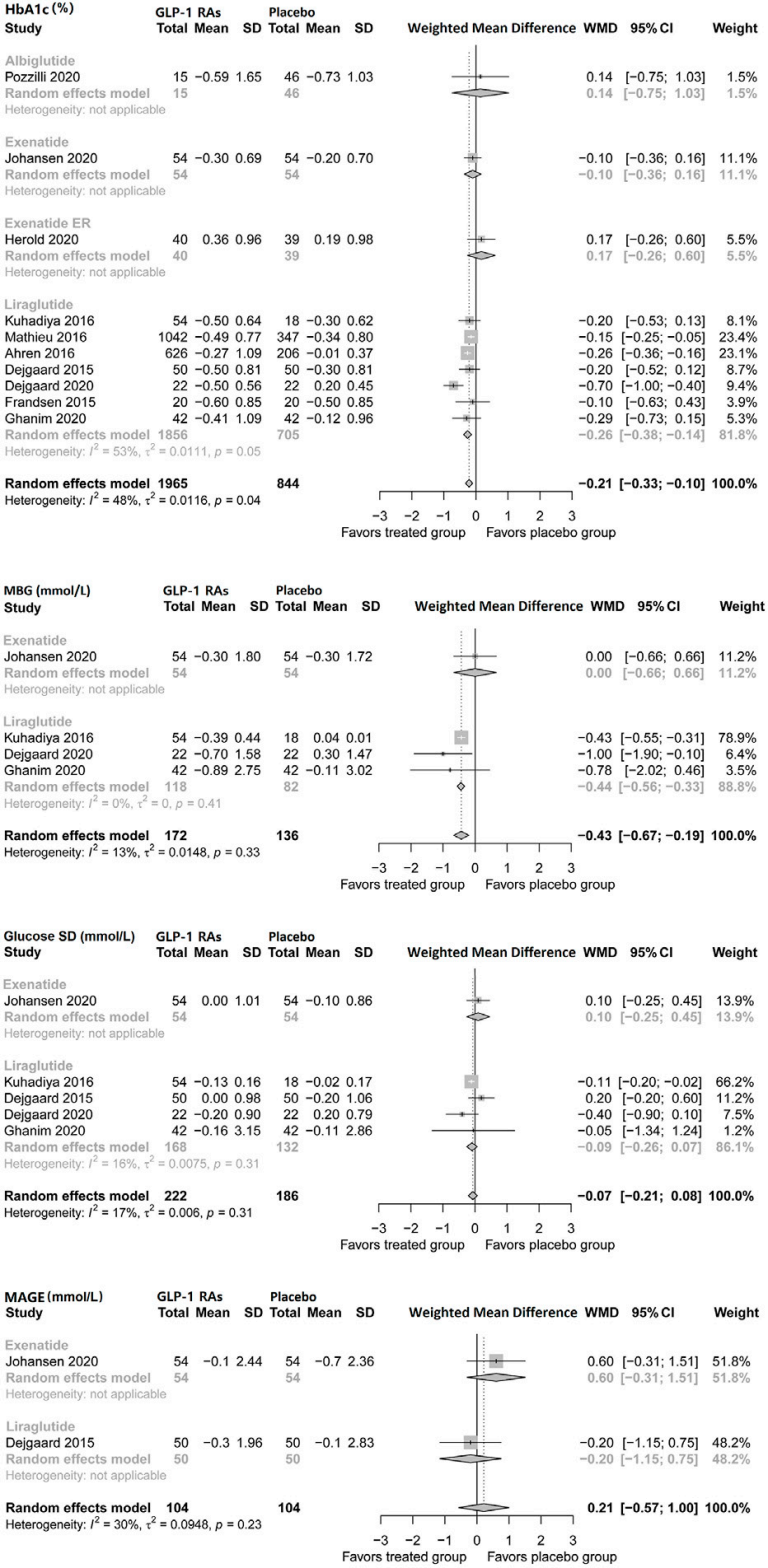
Pairwise meta-analysis comparing the glycemic efficacy of GLP-1 RAs therapy for type 1 diabetes.GLP-1 RAs, Glucagon-like peptide-1 receptor agonists; SD, Standard deviation; CL, Confidence interval; MBG, Mean blood glucose; MAGE, Mean amplitude of glycemic excursions.

Regarding mean blood glucose (MBG), pooled analysis showed no difference, but liraglutide reduced MBG by −0.44 mmol/L (95% CI, −0.56 to −0.33) in patients with type 1 diabetes. No difference was found regarding mean glucose SD. Only two studies (Lira-1 and MAG1C) assessed the mean amplitude of glycemic excursions (MAGE) during treatment, and no difference was found (Figure 1).

#### Insulin dosage

**FIGURE 2:**
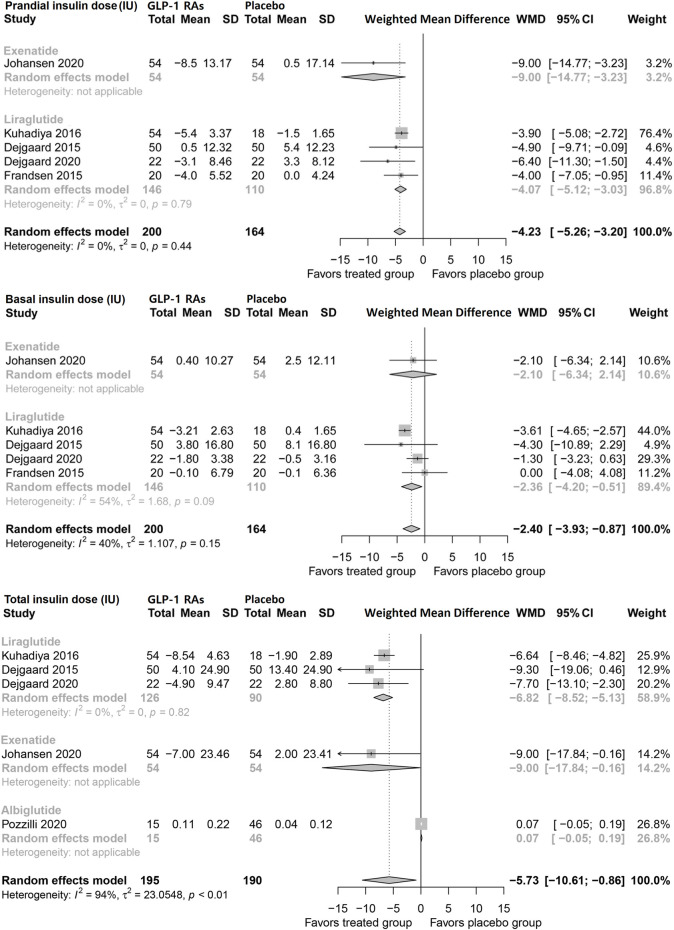
Pairwise meta-analysis comparing the insulin dose of GLP-1 RAs therapy for type 1 diabetes. GLP-1 RAs, Glucagon-like peptide-1 receptor agonists; SD, Standard deviation; CL, Confidence interval.

Pooled analysis showed that GLP-1 RAs reduced prandial insulin dose (WMD, −4.23 IU; 95% CI, −5.26 to −3.20), as well as basal insulin dose (−2.40 IU, −3.93 to −0.87) in patients with type 1 diabetes. Notably, liraglutide reduced basal insulin (2.36 IU; −4.20 to −0.51), but exenatide did not show a significant impact. Consistently, the pooled analysis showed that GLP-1 RAs decreased the total insulin dose (−5.73 IU; −10.61 to −0.86). Both liraglutide and exenatide led to significant reductions in the total insulin dose (Figure 2).

#### Bodyweight

**FIGURE 3:**
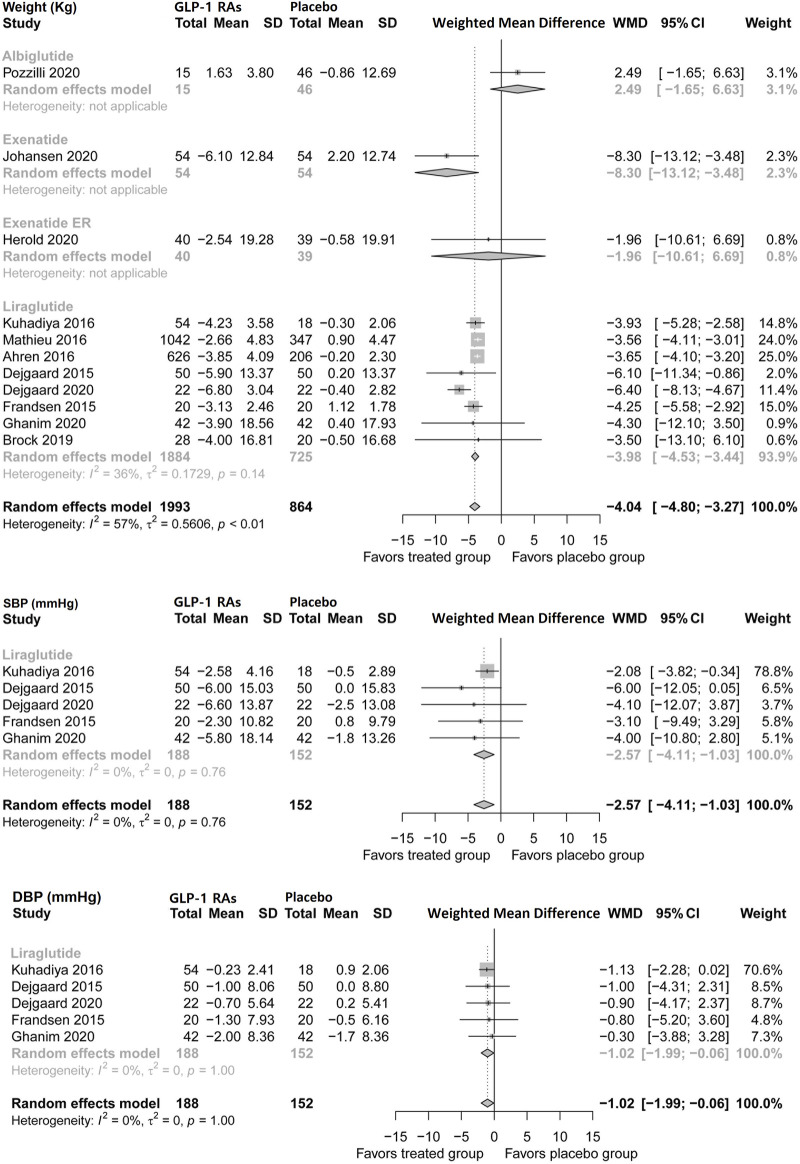
Pairwise meta-analysis comparing the bodyweight and blood pressure of GLP-1 RAs therapy for type 1 diabetes. GLP-1 RAs, Glucagon-like peptide-1 receptor agonists; SD, Standard deviation; CL, Confidence interval; SBP, Systolic blood pressure; DBP, Diastolic blood pressure.

Pooled analysis showed that GLP-1 RAs reduced body weight by 4.04 kg (95% CI, −4.8 to −3.27) in patients with type 1 diabetes. Liraglutide and exenatide reduced body weight by 3.98 kg (−4.54 to −3.44) and 8.3 kg (−13.12 to −3.46), respectively. Albiglutide and exenatide ER did not show favorable effects (Figure 3). This study found no publication bias in this analysis (*p* = 0.7050).

#### Blood pressure

Five trials of liraglutide (n = 340) assessed systolic and diastolic blood pressure during treatment in patients with type 1 diabetes (Figure 3). Liraglutide modestly reduced systolic blood pressure by 2.57 mmHg (95% CI, −4.11 to −1.03) and diastolic blood pressure by 1.02 mmHg (−1.99 to −0.06).

### Safety outcomes

#### Diabetic ketoacidosis

**FIGURE 4:**
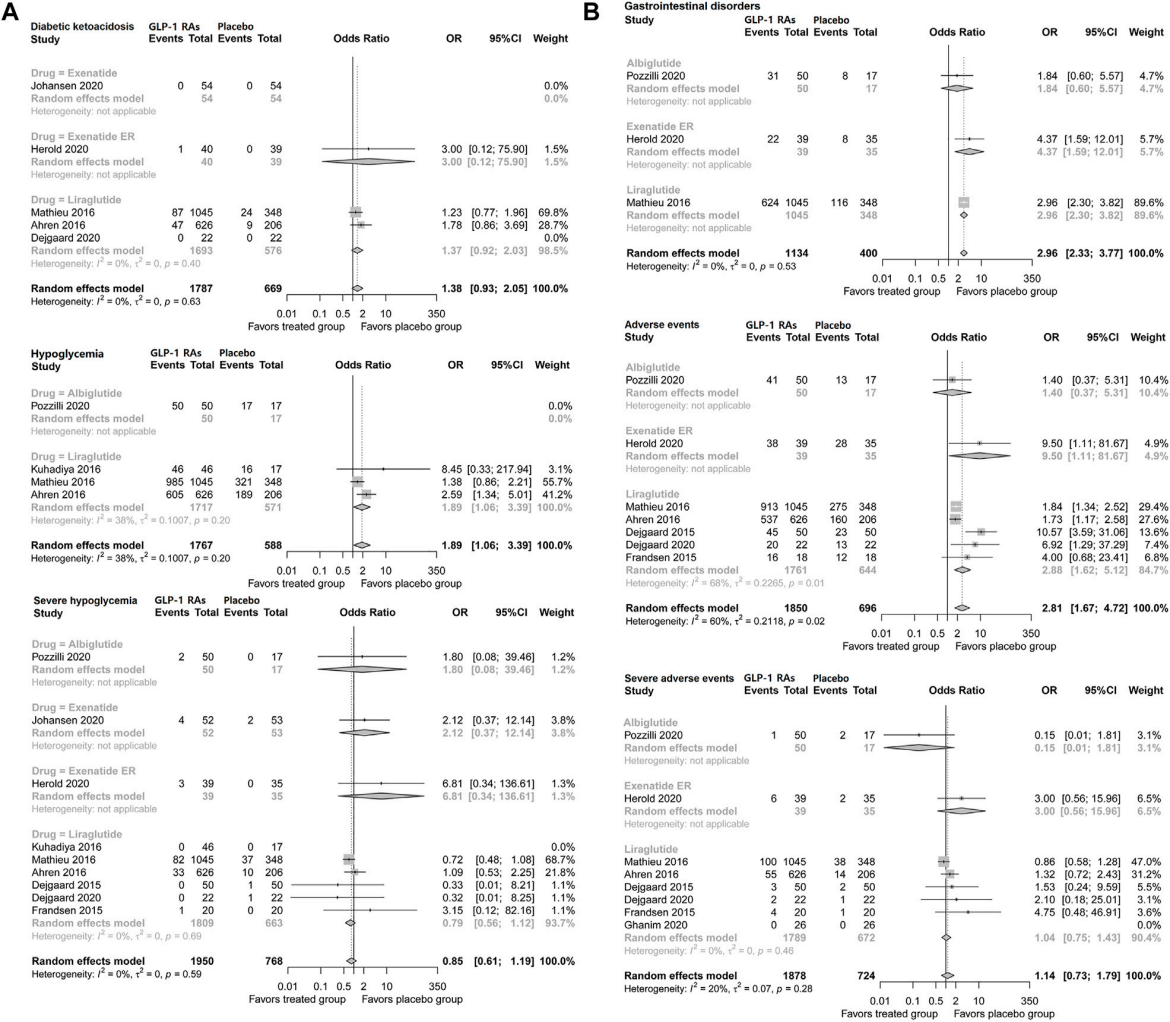
Pairwise meta-analysis comparing the safety of GLP-1 RAs therapy for type 1 diabetes. **(A)** Pairwise meta-analysis comparing the diabetic ketoacidosis and hypoglycemia of GLP-1 RAs therapy for type 1 diabetes. **(B)** Pairwise meta-analysis comparing the gastrointestinal disorders and adverse events of GLP-1 RAs therapy for type 1 diabetes. GLP-1 RAs, Glucagon-like peptide-1 receptor agonists; CL, confidence interval.

Incidence of diabetic ketoacidosis (DKA) was not increased with GLP-1 adjunctive therapy (OR, 1.38; 95% CI, 0.93–2.05) in patients with type 1 diabetes. Liraglutide did not increase the incidence of DKA (1.37; 0.92–2.03), and no difference was found based on the data from the single trial of exenatide ER (Figure 4A).

#### Hypoglycemia

GLP-1 adjunctive therapy was associated with a higher risk of hypoglycemia (OR, 1.89; 95% CI, 1.06–3.39) in patients with type 1 diabetes. However, GLP-1 RA therapy did not increase the risk of severe hypoglycemia (0.86; 0.61–1.19). Consistently, respective analyses of liraglutide, exenatide daily, exenatide ER, and albiglutide did not suggest a higher risk of severe hypoglycemia (Figure 4A).

#### Gastrointestinal disorders

GLP-1 adjunctive therapy significantly increased the incidence of gastrointestinal disorders (OR, 2.96, 95% CI, 2.33–3.77) in patients with type 1 diabetes. Analysis of individual drugs based on the single trial suggested that the incidence of gastrointestinal disorders was higher with liraglutide (2.96; 2.30–3.82) and exenatide ER (4.37; 1.59–12.01) (Figure 4B).

#### Adverse events

Pooled analysis suggested that GLP-1 RA therapy was associated with a higher risk of adverse events (OR, 2.81; 95% CI, 1.67–4.72) in patients with type 1 diabetes. Liraglutide increased the risk of adverse events (2.88; 1.62–5.12), and the results of exenatide ER from a single trial also showed higher risk (9.50; 1.11–81.67), while albiglutide did not show a significant difference. However, GLP-1 RAs did not increase the incidence of severe adverse events (1.14; 0.73–1.79) in patients with type 1 diabetes. No significant difference was found regarding severe adverse events of liraglutide, albiglutide, or exenatide (Figure 4B).

### Subgroup analysis

This study analyzed the impact of liraglutide in obese and overweight patients *versus* normal-weight patients. The HbA1c reduction was −0.43% (95% CI, −0.63 to −0.23) among obese or overweight patients, and −0.10% (−0.63 to 0.43) among normal-weight patients. The weight reduction was −6.28 kg (−7.89 to −4.67) among obese or overweight patients, and −4.25 kg (−5.58 to −2.92) among normal-weight patients. Neither subgroup showed a significant effect of liraglutide on severe hypoglycemia. This study also analyzed the impact of liraglutide in C-peptide-positive *versus* negative patients. The HbA1c reduction amplitude and incidence of DKA was similar in this subgroup analysis. Moreover, the HbA1c reduction was −0.29% (95% CI, −0.37 to −0.21) with long-acting GLP-1 RA therapy and −0.15% (−0.34 to 0.04) with short-acting GLP-1 RA therapy in patients with type 1 diabetes.

## Discussion

In this meta-analysis of 11 up-to-date randomized clinical trials, GLP-1 RA and insulin combination therapy resulted in a modest improvement of metabolic profile, including slight HbA1c reduction, weight loss, a lower insulin dose, and lower blood pressure in patients with type 1 diabetes. Moreover, GLP-1 RAs did not increase the incidence of severe hypoglycemia, diabetic ketoacidosis, or severe adverse events. However, an increased incidence of gastrointestinal events was suggested.

Our meta-analysis showed that GLP-1 RAs modestly but significantly decreased HbA1c levels by 0.21%. In fact, liraglutide is the only GLP-1 RA with improved glycemic control. Seven studies, varying in duration and dosage, assessed liraglutide as an add-on therapy in patients with type 1 diabetes, and three of these trials showed improved glycemic control in patients with combination therapy. Notably, though differing in insulin management strategy, both ADJUNCT ONE and ADJUNCT TWO studies, similarly using 0.6, 1.2, 1.8 mg liraglutide, showed a greater HbA1c reduction at a higher dose during therapy. However, regarding time dependency, the glucose-lowering efficacy initially increased but progressively waned over time in ADJUNCT ONE, ADJUNCT TWO, and Lira-1 studies (Ahrén et al., 2016; Dejgaard et al., 2016; Mathieu et al., 2016). No outcome data were reported about whether the HbA1c reduction would be sustained beyond 1 year of therapy (Doggrell, 2018). Given the chronic course of type 1 diabetes, long-term outcome data of this combination therapy are required.

Thisstudy found that GLP-1 RAs reduced the prandial, basal, and total insulin dose. Findings from Lira-1 and Lira-pump suggest that insulin reduction might be the consequence of weight loss since this effect did not persist after adjusting for bodyweight in patients with type 1 diabetes. On the other hand, GLP-1 RAs increase satiety and decrease food intake, which also affects the bolus insulin dose and postprandial glucose excursions (Dejgaard et al., 2019). Moreover, there might be some different mechanisms for short-acting GLP-1 RAs. In the MAG1c trial, exenatide significantly reduced bolus insulin even after weight adjustment, possibly due to delayed gastric emptying and suppression of postprandial glucagon (Ghazi et al., 2014).

Overall, GLP-1 RAs induced weight loss in patients with type 1 diabetes. GLP-1 RAs counteract insulin-induced weight gain, making combination therapy more beneficial for those patients with obesity (Petrie, 2019). Of note, the result of albiglutide on weight was not directionally concordant with other GLP-1 RAs. It has been proved that the large molecular size of Albiglutide limits its penetration into the brain to induce satiety (Brønden et al., 2017). Our meta-analysis also found that GLP-1 RAs slightly but significantly decreased systolic and diastolic blood pressure. There were doubts about whether this effect is driven by weight loss or is based on the pharmacologic action on GLP-1 receptor blood vessels (Helmstädter et al., 2021). The beneficial effects of GLP-1 RAs on glycemic control, blood pressure, and weight raise the possibility of further cardio-protection (i.e., cardiovascular death, stroke, myocardial infarction, and microvascular complications) in some subgroups of patients with type 1 diabetes (Rawshani et al., 2017; Rawshani et al., 2018). This hypothesis, however, needs further investigation.

Our meta-analysis showed that GLP-1 RAs did not increase the risk of diabetic ketoacidosis in patients with type 1 diabetes. SGLT2 inhibitors, another class of anti-diabetic drugs, also once considered to be used as an add-on treatment, were not approved by FDA to be prescribed in patients with type 1 diabetes due to additional ketoacidosis risks (Henry et al., 2015; Garg et al., 2017; Buse et al., 2018; Dandona et al., 2018; Mathieu et al., 2018; Rosenstock et al., 2018; Taylor et al., 2019). However, the European Commission has authorized dapagliflozin and sotagliflozin as add-on treatments for type 1 diabetes (First oral add-on treatment to insulin for treatment 2019; New add-on treatment to insulin for treatment, 2019). Clinicians should take careful consideration before combining GLP-1RA with SGLT-2 inhibitor in type 1 diabetes, and intensive monitoring of β-hydroxybutyrate concentrations is highly recommended in these cases (Kuhadiya et al., 2016). Despite the increased risk of hypoglycemia, GLP-1 RAs did not increase the risk of severe hypoglycemia. Similarly, although there were more adverse events for GLP-1 RA combination therapy, the incidence of severe adverse events did not increase. As seen in patients with T2D, GLP-1 RAs increased the incidence of gastrointestinal events (Eng et al., 2014). Taken together, GLP-1 RAs showed a generally safe benefit-to-risk profile in patients with type 1 diabetes.

Obese or overweight *versus* normal-weight patients merit further discussion. Four studies specified the weight status of the participants, and our meta-analysis suggested that GLP-1 RAs have greater effects on HbA1c and bodyweight reduction in the obese or overweight group. However, a recent *post-hoc* analysis of ADJUNCT ONE and ADJUNCT TWO suggested that the baseline level of BMI did not impact the safety or efficacy outcomes of liraglutide in type 1 diabetes (Dejgaard et al., 2021). In ADJUNCT ONE trial, insulin adjustment followed the treat-to-target strategy. Namely, the insulin dose was adjusted to reach target postprandial glucose levels. In the ADJUNCT TWO trial, the insulin dose was adjusted within the limits of the insulin cap, which was defined as the average of the previous seven consecutive days’ total daily insulin dose. Similarly, both ADJUNCT ONE and ADJUNCT TWO trials reduced 25% of the total daily insulin dose on the day of randomization and a further 10% on subsequent days of liraglutide dose escalation.

Compared with short-acting GLP-1 RAs, our subgroup analysis showed a greater reduction in HbA1c with long-acting GLP-1 RAs. Similar findings were also observed in two head-to-head clinical trials in patients with type 2 diabetes (Drucker et al., 2008; Buse et al., 2009). This is likely due to different pharmacokinetic profiles, indicating that sustained GLP-1 receptor activation results in greater suppression of fasting glucagon.

Recently, two network meta-analyses, both including GLP-1 RAs, assessed available antidiabetic medications as an add-on therapy in patients with type 1 diabetes (Kim et al., 2020; Avgerinos et al., 2021). Besides the different analysis methodology and scope, our present pair-wise analysis differed from theirs in the following ways. First, these two studies both included trails shorter than 8 weeks. Our meta-analysis excluded these trails, since such a short duration was not long enough to achieve glycemic steady state. Second, both network analyses included cross-over studies, which may have increased unit-of-analysis errors and bias for carry-over effects. To avoid this, this meta-analysis only included evidence from prospective randomized controlled trials to ensure the quality of the meta-analysis. Third, Ioannis Avgerinos et al. did not include RCTs with albiglutide and exenatide ER, but their conclusion of the main efficacy and safety outcomes was concordant with ours. Yoon Ji Kim et al. involved fewer patients from six studies (four liraglutide and two exenatide) with GLP-1 RAs, and did not detect significant differences regarding HbA1c, insulin dose, and blood pressure.

Several limitations of this analysis should be acknowledged. First, the long-term impact of GLP-1 RAs on patients with type 1 diabetes is unknown; there is no evidence beyond 1 year (Table 1). The effects of GLP-1RA on weight and glycemic control might be mitigated with prolonged therapy. Second, although most of the included studies were of high-quality, they carry a potential risk of bias due to pharmaceutical industry funding. Third, the appropriate timing for beginning this combination therapy in the course of type 1 diabetes remains unknown. Recently, two trials suggest that liraglutide preserve beta-cell function in patients with newly diagnosed type 1 diabetes (von Herrath et al., 2021). Therefore, it is speculated that earlier implementation of this combination therapy might be helpful. Given the minor effects of GLP-1 RAs on glycemic control, it is important for clinicians to evaluate the benefits and risks in real-world practice.

## Conclusion

In conclusion, our up-to-date meta-analysis suggests modest beneficial effects of GLP-1 RAs on the metabolic profile in patients with type 1 diabetes, without increased risks of DKA, severe hypoglycemia, or severe adverse events. Given the lack of long-term evidence, careful evaluation should be made before prescribing this class of agents in real-world practice.

## Data Availability

The original contributions presented in the study are included in the article/Supplementary Material, further inquiries can be directed to the corresponding authors.
