# Supplementary material for: Glucagon-like peptide-1 receptor agonists as add-on therapy to insulin for type 1 diabetes mellitus
Source: Front Pharmacol. 2023 Mar 16;14:975880. doi: 10.3389/fphar.2023.975880 (PMC10797415; doi:10.3389/fphar.2023.975880)
Supplement: Supplementary file 1 [file DataSheet1.zip › Appendix 2. Protocal GLP-1 prospero.pdf]

National Institute for Health Research

UNIVERSITY of York
Centre for Reviews and Dissemination

Systematic review

1. * Review title.

Give the title of the review in English

Glucagon-like peptide-1 receptor agonists as add-on therapy to insulin for type 1 diabetes mellitus:

systematic review and meta-analysis of randomized controlled trials

2. Original language title.

For reviews in languages other than English, give the title in the original language. This will be displayed with the English language title.

3. * Anticipated or actual start date.

Give the date the systematic review started or is expected to start.

01/05/2020

4. * Anticipated completion date.

Give the date by which the review is expected to be completed.

31/10/2020

5. * Stage of review at time of this submission.

Tick the boxes to show which review tasks have been started and which have been completed. Update this field each time any amendments are made to a published record.

Reviews that have started data extraction (at the time of initial submission) are not eligible for inclusion in PROSPERO. If there is later evidence that incorrect status and/or completion date has been supplied, the published PROSPERO record will be marked as retracted.

This field uses answers to initial screening questions. It cannot be edited until after registration.

The review has not yet started: No

Review stage	Started	Completed
Preliminary searches	Yes	Yes
Piloting of the study selection process	Yes	Yes
Formal screening of search results against eligibility criteria	Yes	No
Data extraction	No	No
Risk of bias (quality) assessment	No	No
Data analysis	No	No

Provide any other relevant information about the stage of the review here.

6. * Named contact.

The named contact is the guarantor for the accuracy of the information in the register record. This may be any member of the review team.

Xinrui Tan

Email salutation (e.g. "Dr Smith" or "Joanne") for correspondence:

Dr Tan

7. * Named contact email.

Give the electronic email address of the named contact.

xinruitan@csu.edu.cn

8. Named contact address

Give the full institutional/organisational postal address for the named contact.

Renming Road No.139, Changsha, Hunan 410011, China,

9. Named contact phone number.

Give the telephone number for the named contact, including international dialling code.

+86 13397618602

10. * Organisational affiliation of the review.

Full title of the organisational affiliations for this review and website address if available. This field may be completed as 'None' if the review is not affiliated to any organisation.

the Second Xiangya Hospital of Central South University

Organisation web address:

11. * Review team members and their organisational affiliations.

Give the personal details and the organisational affiliations of each member of the review team. Affiliation refers to groups or organisations to which review team members belong. **NOTE: email and country now MUST be entered for each person, unless you are amending a published record.**

Dr Xinrui Tan. the Second Xiangya Hospital of Central South University
Xingxing Zhang. the Second Xiangya Hospital of Central South University
Xiaochuan Wu. the Second Xiangya Hospital of Central South University

12. * Funding sources/sponsors.

Details of the individuals, organizations, groups, companies or other legal entities who have funded or sponsored the review.

Provincial Natural Science Fund of Hunan

Grant number(s)

State the funder, grant or award number and the date of award

2019JJ50897

13. * Conflicts of interest.

List actual or perceived conflicts of interest (financial or academic).

None

14. Collaborators.

Give the name and affiliation of any individuals or organisations who are working on the review but who are not listed as review team members. **NOTE: email and country must be completed for each person, unless you are amending a published record.**

15. * Review question.

State the review question(s) clearly and precisely. It may be appropriate to break very broad questions down into a series of related more specific questions. Questions may be framed or refined using PI(E)COS or similar where relevant.

People with type 1 diabetes need intensive insulin therapy for glycemic control. However, this is quite challenging due to the risks of hypoglycemia and weight gain. GLP-1 receptor agonists (GLP-1 RAs) is a class of anti-diabetic drugs derived from incretin hormone GLP-1, which stimulate glucose-dependent insulin secretion, inhibit glucagon secretion, increase satiety, and slow gastrointestinal motility. It has been demonstrated that GLP-1 receptor agonists improve glycemic control and induce weight loss in subjects with type 2 diabetes. Combination therapy with insulin and GLP-1RAs also seems promising for type 1 diabetes management. However, evidence from randomized controlled trials of such combination therapy is inconsistent. Whether GLP-1 receptor agonists could safely improve glycemic control in type 1 diabetes is uncertain.

16. * Searches.

State the sources that will be searched (e.g. Medline). Give the search dates, and any restrictions (e.g. language or publication date). Do NOT enter the full search strategy (it may be provided as a link or attachment below.)

The following databases were searched from inception to 15 July 2020: PubMed, Embase, and Cochrane

Central Register of Controlled Trials.

17. URL to search strategy.

Upload a file with your search strategy, or an example of a search strategy for a specific database, (including the keywords) in pdf or word format. In doing so you are consenting to the file being made publicly accessible. Or provide a URL or link to the strategy. Do NOT provide links to your search **results**.

https://www.crd.york.ac.uk/PROSPEROFILES/199840_STRATEGY_20200722.pdf

Alternatively, upload your search strategy to CRD in pdf format. Please note that by doing so you are consenting to the file being made publicly accessible.

Do not make this file publicly available until the review is complete

18. * Condition or domain being studied.

Give a short description of the disease, condition or healthcare domain being studied in your systematic review.

Type 1 diabetes is an autoimmune disease characterized by chronic hyperglycemia due to absolute endogenous insulin deficiency. There is no cure for type 1 diabetes, and patients need intensive insulin treatment for blood glucose control. If not well controlled, it will lead to diabetes-related complications, including cardiovascular diseases, nephropathy, neuropathy, etc.

19. * Participants/population.

Specify the participants or populations being studied in the review. The preferred format includes details of both inclusion and exclusion criteria.

Key inclusion criteria comprised the following: type 1 diabetes duration ?1 year, age ?12 years, treatment with multiple daily injections (MDI) of insulin or continuous subcutaneous insulin infusion (CSII) for at least 6 months, with a stable insulin dose for at least 3 months. Key exclusion criteria comprised the following: any prior use of GLP-1RAs or dipeptidyl peptidase-4 inhibitors, any medication (except insulin) that could interfere with glycemic control or affect a subject's safety, or an estimated glomerular filtration rate 30 mL/min/1.73 m2, history of acute or chronic pancreatitis, other relevant medical conditions as judged by the investigator.

20. * Intervention(s), exposure(s).

Give full and clear descriptions or definitions of the interventions or the exposures to be reviewed. The preferred format includes details of both inclusion and exclusion criteria.

Glucagon-Like Peptide-1 Receptor Agonists (GLP-1 RAs) were developed based on incretin hormone GLP-1. These agents, with longer duration compared to native GLP-1, stimulate glucose-mediated insulin secretion, inhibit glucagon secretion, promote satiety, and delay gastric emptying. GLP-1 RAs improve glycemic control with favorable safety profiles in patients with type 2 diabetes. These agents also have positive effects on body weight, blood pressure, cholesterol levels, and beta-cell function.

Currently, there are seven GLP-1 RAs available for treatment. They are categorized as short-acting and long-

acting compounds according to their chemical structure and pharmacokinetic profile. Exenatide and lixisenatide are short-acting GLP-1 RAs that are based on an exendin 4 structure (isolated from the saliva of the lizard Heloderma suspectum), and exenatide once weekly is the only long-acting GLP-1 RA structurally based on exendin 4, whereas other long-acting GLP-1 RAs (liraglutide, dulaglutide, albiglutide and semaglutide) are all based on the GLP1 structure.

21. * Comparator(s)/control.

Where relevant, give details of the alternatives against which the intervention/exposure will be compared (e.g. another intervention or a non-exposed control group). The preferred format includes details of both inclusion and exclusion criteria.

In this meta-analysis, RCTs comparing GLP-1 RAs plus insulin versus placebo plus insulin in subjects with type 1 diabetes were included. Placebo was administered in the same way as GLP-1 RAs at corresponding volume.

22. * Types of study to be included.

Give details of the study designs (e.g. RCT) that are eligible for inclusion in the review. The preferred format includes both inclusion and exclusion criteria. If there are no restrictions on the types of study, this should be stated.

Studies were included in the meta-analysis if they: (1) were prospective, randomized, and controlled clinical trials; (2) assess the efficacy and safety of GLP-1 RAs as adjunct therapy in patients with type 1 diabetes; (3) at least 8 weeks of intervention. Studies were excluded if they:(1) were case reports, retrospective or cross-over studies; (2) did not report HbA1c, body weight and total daily insulin dose at baseline and end of trial; (3) if they included special populations (e.g. individuals with gestational diabetes), or if they included patients with type 2 diabetes.

23. Context.

Give summary details of the setting or other relevant characteristics, which help define the inclusion or exclusion criteria.

24. * Main outcome(s).

Give the pre-specified main (most important) outcomes of the review, including details of how the outcome is defined and measured and when these measurement are made, if these are part of the review inclusion criteria.

Main outcomes of interest included efficacy variables (HbA1c (%), fasting plasma glucose (FPG) (mmol/l), mean blood glucose(mmol/l) determined by continuous glucose monitoring (CGM), standard deviation of glucose by CGM, and mean amplitude of glucose excursions by CGM , weight, total insulin dose (units/day), bolus insulin dose (units/day), and basal insulin dose (units/day)) and safety variables (hypoglycemia, severe hypoglycemia, diabetic ketoacidosis).

* Measures of effect

Please specify the effect measure(s) for you main outcome(s) e.g. relative risks, odds ratios, risk difference,

and/or 'number needed to treat.

Weighted mean difference (WMD) was calculated by DerSimonian-Laird random effects meta-analysis to evaluate the influence of GLP-1RAs on HbA1c, body weight, fasting plasma glucose, mean blood glucose, BMI, total insulin dose, bolus insulin dose, basal insulin dose and other continuous variables.

Pooled odds ratio (OR) were calculated by DerSimonian-Laird random effects meta-analysis to evaluate the influence of GLP-1RAs on safety events (hypoglycemia, severe hypoglycemia, diabetic ketoacidosis) and other Dichotomous variables.

25. * Additional outcome(s).

List the pre-specified additional outcomes of the review, with a similar level of detail to that required for main outcomes. Where there are no additional outcomes please state 'None' or 'Not applicable' as appropriate to the review

Additional outcomes of interest included efficacy variables (systolic and diastolic blood pressure(mmHg), carbohydrate intake(g), heart rates) and safety variables (gastrointestinal adverse events, such as nausea, diarrhea, vomiting).

* Measures of effect

Please specify the effect measure(s) for you additional outcome(s) e.g. relative risks, odds ratios, risk difference, and/or 'number needed to treat.

Weighted mean difference (WMD) was calculated by DerSimonian-Laird random effects meta-analysis to evaluate the influence of GLP-1RAs on blood pressure, carbohydrate intake, and heart rates.

Pooled odds ratio (OR) were calculated by DerSimonian-Laird random effects meta-analysis to evaluate the influence of GLP-1RAs on gastrointestinal adverse events (nausea, diarrhea, vomiting).

26. * Data extraction (selection and coding).

Describe how studies will be selected for inclusion. State what data will be extracted or obtained. State how this will be done and recorded.

Data were extracted by two independent authors, with conflicts over study inclusion resolved by consensus. We extracted the baseline demographic data, study duration, type of GLP-1 RA used, and outcomes of each trial. Investigators who performed the studies were contacted by e-mail to obtain missing information as needed. Data from multiple reports on the same study were collated. If more than one study covered the same population, only the report containing the most comprehensive information was analyzed to avoid overlapping populations. When published studies reported outcomes for various follow-up periods, data from the primary outcome follow-up period were employed for efficacy analysis, while the longest follow-up period was used to assess adverse outcomes (hypoglycemia, etc).

27. * Risk of bias (quality) assessment.

State which characteristics of the studies will be assessed and/or any formal risk of bias/quality assessment tools that will be used.

The risk of bias of included studies was assessed independently by two reviewers through the Cochrane risk-of bias tool for the following aspects: random sequence generation; allocation concealment; blinding of participants and personnel; blinding of outcome assessment; incomplete outcome data; selecting reporting. For other bias, funding and authorship were assessed. Each domain was assigned low, unclear, or high risk of bias.

28. * Strategy for data synthesis.

Describe the methods you plan to use to synthesise data. This **must not be generic text** but should be **specific to your review** and describe how the proposed approach will be applied to your data. If meta-analysis is planned, describe the models to be used, methods to explore statistical heterogeneity, and software package to be used.

For each outcome of interest, the mean difference (MD) and its 95% CI were applied for continuous variables (HbA1c, weight, insulin dose, etc) while odds ratio (OR) and its 95% CI were used for dichotomous outcomes (episode of hypoglycemia). Considering differences in baseline participants' characteristics and drug administration, a random effects model was selected for analyses. A P value less than .05 for any test or model was considered statistically significant.

The degree of between?study variability attributable to heterogeneity beyond chance was calculated using the I^2 statistic and Q statistic. Outcomes with I^2 levels from 0% to 40% were considered minimally heterogeneous, while I^2 50% was considered an indication of statistically significant heterogeneity among included studies. We conducted subgroup analyses and sensitivity analyses to address the heterogeneity of included studies. Forest plots were used for graphic representation of the data. Funnel plots and Egger test were used for assessing publication bias.

Risk of bias assessment was performed by the Review Manager statistical software package (Version 5.3), and the meta?analyses and regression?analyses were performed by the STATA statistical software package (Version 12.0).

29. * Analysis of subgroups or subsets.

State any planned investigation of ‘subgroups’. Be clear and specific about which type of study or participant will be included in each group or covariate investigated. State the planned analytic approach.

Subgroup analyses based on different types of GLP-1 RAs.

30. * Type and method of review.

Select the type of review, review method and health area from the lists below.

Type of review

Cost effectiveness
No

Diagnostic
No

Epidemiologic
No

Individual patient data (IPD) meta-analysis
No

Intervention
No

Meta-analysis
Yes

Methodology
No

Narrative synthesis
No

Network meta-analysis
No

Pre-clinical
No

Prevention
No

Prognostic
No

Prospective meta-analysis (PMA)
No

Review of reviews
No

Service delivery
No

Synthesis of qualitative studies
No

Systematic review
Yes

Other
No

Health area of the review

Alcohol/substance misuse/abuse
No

Blood and immune system
No

Cancer
No

Cardiovascular

No

Care of the elderly
No

Child health
No

Complementary therapies
No

COVID-19
No

Crime and justice
No

Dental
No

Digestive system
No

Ear, nose and throat
No

Education
No

Endocrine and metabolic disorders
Yes

Eye disorders
No

General interest
No

Genetics
No

Health inequalities/health equity
No

Infections and infestations
No

International development
No

Mental health and behavioural conditions
No

Musculoskeletal
No

Neurological
No

Nursing
No

Obstetrics and gynaecology
No

Oral health
No

Palliative care
No

Perioperative care
No

Physiotherapy
No

Pregnancy and childbirth
No

Public health (including social determinants of health)
No

Rehabilitation
No

Respiratory disorders
No

Service delivery
No

Skin disorders
No

Social care
No

Surgery
No

Tropical Medicine
No

Urological
No

Wounds, injuries and accidents
No

Violence and abuse
No

31. Language.

Select each language individually to add it to the list below, use the bin icon to remove any added in error.
English

There is not an English language summary

32. * Country.

Select the country in which the review is being carried out. For multi-national collaborations select all the countries involved.

China

33. Other registration details.

Name any other organisation where the systematic review title or protocol is registered (e.g. Campbell, or The Joanna Briggs Institute) together with any unique identification number assigned by them. If extracted data will be stored and made available through a repository such as the Systematic Review Data Repository (SRDR), details and a link should be included here. If none, leave blank.

34. Reference and/or URL for published protocol.

If the protocol for this review is published provide details (authors, title and journal details, preferably in Vancouver format)

Add web link to the published protocol.

Or, upload your published protocol here in pdf format. Note that the upload will be publicly accessible.

No I do not make this file publicly available until the review is complete

Please note that the information required in the PROSPERO registration form must be completed in full even if access to a protocol is given.

35. Dissemination plans.

Do you intend to publish the review on completion?

Yes

Give brief details of plans for communicating review findings.?

36. Keywords.

Give words or phrases that best describe the review. Separate keywords with a semicolon or new line. Keywords help PROSPERO users find your review (keywords do not appear in the public record but are included in searches). Be as specific and precise as possible. Avoid acronyms and abbreviations unless these are in wide use.

37. Details of any existing review of the same topic by the same authors.

If you are registering an update of an existing review give details of the earlier versions and include a full bibliographic reference, if available.

38. * Current review status.

Update review status when the review is completed and when it is published.New registrations must be ongoing so this field is not editable for initial submission.
Please provide anticipated publication date

Review_Ongoing

39. Any additional information.

Provide any other information relevant to the registration of this review.

40. Details of final report/publication(s) or preprints if available.

Leave empty until publication details are available OR you have a link to a preprint (NOTE: this field is not editable for initial submission). List authors, title and journal details preferably in Vancouver format.

Give the link to the published review or preprint.
