# Supplementary material for: Glucagon-like peptide-1 receptor agonists as add-on therapy to insulin for type 1 diabetes mellitus
Source: Front Pharmacol. 2023 Mar 16;14:975880. doi: 10.3389/fphar.2023.975880 (PMC10797415; doi:10.3389/fphar.2023.975880)
Supplement: Supplementary file 1 [file DataSheet1.zip › Appendix 3. GLP-1 T1DM Search Strategy.docx]

**Appendix 3. GLP-1 T1DM Search Strategy**

Medline (Pubmed) (1950- 3 July 2020)

| #1 | Glucagon-Like Peptide 1[MeSH Terms] |
| --- | --- |
| #2 | Glucagon Like Peptide 1[Title/Abstract] |
| #3 | GLP-1[Title/Abstract] |
| #4 | GLP 1[Title/Abstract] |
| #5 | Glucagon-Like Peptide-1[Title/Abstract] |
| #6 | exenatide[Title/Abstract] |
| #7 | lixisenatide[Title/Abstract] |
| #8 | liraglutide[Title/Abstract] |
| #9 | albiglutide[Title/Abstract] |
| #10 | dulaglutide[Title/Abstract] |
| #11 | semaglutide[Title/Abstract] |
| #12 | #1 OR #2 OR #3 OR #4 OR #5 OR #6 OR #7 OR #8 OR #9 OR #10 OR #11 |
| #13 | (Diabetes Mellitus, Type 1[MeSH Terms]) |
| #14 | Type 1 Diabetes[Title/Abstract] |
| #15 | Insulin-Dependent Diabetes Mellitus 1[Title/Abstract] |
| #16 | IDDM[Title/Abstract] |
| #17 | Autoimmune Diabetes[Title/Abstract] |
| #18 | Brittle Diabetes Mellitus[Title/Abstract] |
| #19 | Ketosis-Prone Diabetes Mellitus[Title/Abstract] |
| #20 | (Diabetes Mellitus, Type 2[MeSH Terms]) OR Type 2 Diabetes[Title] |
| #21 | (#13 OR #14 OR #15 OR #16 OR #17 OR #18 OR #19) NOT #20 |
| #22 | randomized controlled trial[Publication Type] |
| #23 | (random* OR placebo* OR single blind* OR double blind* OR triple blind*) |
| #24 | (retraction of publication[Publication Type] OR retracted publication[Publication Type]) |
| #25 | ((animals not humans)[MeSH Terms]) |
| #26 | (((comment[Publication Type] OR editorial[Publication Type] OR meta-analysis[Publication Type] OR practice-guideline[Publication Type] OR review[Publication Type] OR letter) not randomized controlled trial[Publication Type]) |
| #27 | ((random sampl* or random digit* or random effect* or random survey or random regression)[Title/Abstract] not randomized controlled trial[Publication Type]) |
| #28 | #25 or #26 or #27 |
| #29 | (#22 or #23 or #24) NOT #28 |
| #30 | #12 AND #21 AND #29 |

Embase

(1950- 3 July 2020)

| #1 | 'glucagon like peptide 1'/exp |
| --- | --- |
| #2 | 'glucagon like peptide 1':ab,ti |
| #3 | 'glp-1':ab,ti |
| #4 | 'glp 1':ab,ti |
| #5 | 'glucagon-like peptide-1':ab,ti |
| #6 | 'exenatide'/exp |
| #7 | 'lixisenatide'/exp |
| #8 | 'liraglutide'/exp |
| #9 | 'albiglutide'/exp |
| #10 | 'dulaglutide'/exp |
| #11 | 'semaglutide'/exp |
| #12 | #1 OR #2 OR #3 OR #4 OR #5 OR #6 OR #7 OR #8 OR #9 OR #10 OR #11 |
| #13 | 'insulin dependent diabetes mellitus'/exp |
| #14 | 'type 1 diabetes':ab,ti |
| #15 | 'iddm':ab,ti |
| #16 | 'autoimmune diabetes':ab,ti |
| #17 | 'brittle diabetes mellitus':ab,ti |
| #18 | 'ketosis-prone diabetes mellitus':ab,ti |
| #19 | #13 OR #14 OR #15 OR #16 OR #17 OR #18 |
| #20 | 'clinical trial'/exp OR 'controlled clinical trial'/exp OR 'randomized controlled trial'/exp |
| #21 | random*:ab,ti OR placebo*:ab,ti OR 'single blind*':ab,ti OR 'double blind*':ab,ti OR 'triple blind*':ab,ti |
| #22 | trial*:ab,ti OR control*:ab,ti |
| #23 | 'randomized controlled trial':ti |
| #24 | #20 OR #21 OR #22 OR #23 |
| #25 | #12 AND #19 AND #24 |

Cochrane library (~1950- 3 July 2020)

| #1 | MeSH descriptor: [Glucagon-Like Peptide 1] explode all trees |
| --- | --- |
| #2 | (Glucagon Like Peptide 1):ti,ab,kw |
| #3 | (GLP-1):ti,ab,kw |
| #4 | (GLP 1):ti,ab,kw |
| #5 | (Glucagon-Like Peptide-1):ti,ab,kw |
| #6 | #1 OR #2 OR #3 OR #4 OR #5 |
| #7 | MeSH descriptor: [Exenatide] explode all trees |
| #8 | (lixisenatide):ti,ab,kw |
| #9 | MeSH descriptor: [Liraglutide] explode all trees |
| #10 | (albiglutide):ti,ab,kw |
| #11 | (dulaglutide):ti,ab,kw |
| #12 | (dulaglutide):ti,ab,kw |
| #13 | #6 OR #7 OR #8 OR #9 OR #10 OR #11 OR #12 |
| #14 | MeSH descriptor: [Diabetes Mellitus, Type 1] explode all trees |
| #15 | (Insulin-Dependent Diabetes Mellitus):ti,ab,kw (Word variations have been searched) |
| #16 | (Autoimmune Diabetes):ti,ab,kw |
| #17 | #14 OR #15 OR #16 |
| #18 | MeSH descriptor: [Diabetes Mellitus, Type 2] explode all trees |
| #19 | (Non-insulin dependent Diabetes Mellitus):ti,ab,kw |
| #20 | #18 OR #19 |
| #21 | #17 NOT #20 |
| #22 | #13 AND #21 |
