# Supplementary material for: Glucagon-like peptide-1 receptor agonists as add-on therapy to insulin for type 1 diabetes mellitus
Source: Front Pharmacol. 2023 Mar 16;14:975880. doi: 10.3389/fphar.2023.975880 (PMC10797415; doi:10.3389/fphar.2023.975880)
Supplement: Supplementary file 1 [file DataSheet1.zip › Appendix 4. Definitions of hypoglycemia and severe hypoglycemia.docx]

**Appendix 4. Definitions of hypoglycemia and severe hypoglycemia**

| Study | Hypoglycemia | Severe hypoglycemia | Reference |
| --- | --- | --- | --- |
| Kuhadiya 2016 | <55 mg/dL, 3.05 mmol/L | NR | NR |
| Mathieu 2016 | Documented symptomatic hypoglycemia: It is an event during which typical symptoms of hypoglycemia are accompanied by a measured plasma glucose concentration ≤70 mg/dL (≤3.9 mmol/L). Asymptomatic hypoglycemia: It is an event not accompanied by typical symptoms of hypoglycemia but with a measured plasma glucose concentration ≤70 mg/dL (≤3.9 mmol/L). Probable symptomatic hypoglycemia: It is an event during which symptoms typical of hypoglycemia are not accompanied by a plasma glucose determination but that was presumably caused by a plasma glucose concentration ≤70 mg/dL (≤3.9 mmol/L). Pseudo-hypoglycemia: It is an event during which the person with diabetes reports any of the typical symptoms of hypoglycemia with a measured plasma glucose concentration >70 mg/dL (>3.9 mmol/L) but approaching that level. | Severe hypoglycemia is an event requiring assistance of another person to actively administer carbohydrates, glucagon, or take other corrective actions. Plasma glucose concentrations may not be available during an event, but neurological recovery following the return of plasma glucose to normal is considered sufficient evidence that the event was induced by a low plasma glucose concentration. | ^[1]^ |
| Ahren 2016 | Documented symptomatic hypoglycemia: It is an event during which typical symptoms of hypoglycemia are accompanied by a measured plasma glucose concentration ≤70 mg/dL (≤3.9 mmol/L). Asymptomatic hypoglycemia: It is an event not accompanied by typical symptoms of hypoglycemia but with a measured plasma glucose concentration ≤70 mg/dL (≤3.9 mmol/L). Probable symptomatic hypoglycemia: It is an event during which symptoms typical of hypoglycemia are not accompanied by a plasma glucose determination but that was presumably caused by a plasma glucose concentration ≤70 mg/dL (≤3.9 mmol/L). Pseudo-hypoglycemia: It is an event during which the person with diabetes reports any of the typical symptoms of hypoglycemia with a measured plasma glucose concentration >70 mg/dL (>3.9 mmol/L) but approaching that level. | Severe hypoglycemia is an event requiring assistance of another person to actively administer carbohydrates, glucagon, or take other corrective actions. Plasma glucose concentrations may not be available during an event, but neurological recovery following the return of plasma glucose to normal is considered sufficient evidence that the event was induced by a low plasma glucose concentration. | ^[1]^ |
| Dejgaard 2015 | blood glucose ≤3·9 mmol/L | NR | NR |
| Johansen 2020 | level 1 hypoglycaemia[3·0–3·9 mmol/L]; level 2 hypoglycaemia[<3·0 mmol/L]; | severe hypoglycaemia requiring third party assistance | ^[2]^ |
| Dejgaard 2020 | hypoglycaemia level 1: glucose value <4.0 mmol/L (71 mg/dL); hypoglycaemia level 2: glucose value <3.0 mmol/L (54 mg/dL) | Severe hypoglycemia (level 3): No specific glucose threshold. Hypoglycemia associated with severe cognitive impairment requiring external assistance for recovery | ^[3]^ |
| Pozzilli 2020 | Documented symptomatic hypoglycemia: It is an event during which typical symptoms of hypoglycemia are accompanied by a measured plasma glucose concentration ≤70 mg/dL (≤3.9 mmol/L). Asymptomatic hypoglycemia: It is an event not accompanied by typical symptoms of hypoglycemia but with a measured plasma glucose concentration ≤70 mg/dL (≤3.9 mmol/L). Probable symptomatic hypoglycemia: It is an event during which symptoms typical of hypoglycemia are not accompanied by a plasma glucose determination but that was presumably caused by a plasma glucose concentration ≤70 mg/dL (≤3.9 mmol/L). Pseudo-hypoglycemia: It is an event during which the person with diabetes reports any of the typical symptoms of hypoglycemia with a measured plasma glucose concentration >70 mg/dL (>3.9 mmol/L) but approaching that level. | Severe hypoglycemia is an event requiring assistance of another person to actively administer carbohydrates, glucagon, or take other corrective actions. Plasma glucose concentrations may not be available during an event, but neurological recovery following the return of plasma glucose to normal is considered sufficient evidence that the event was induced by a low plasma glucose concentration. | ^[1]^ |
| Frandsen 2015 | Hypoglycemia (any type): self-monitored blood glucose(SMBG) glucose levels ≤70 mg/dL(≤3.9 mmol/L) | Severe hypoglycemia: any event requiring assistance of another person to actively administer carbohydrates, glucagon, or take other corrective actions with or without measurement of blood glucose levels. | ^[1]^ |
| Herold 2020 | Level 1 between 55 and 70 mg/dl; Level 2 hypoglycemia < 55 mg/dl | Severe hypoglycemia was designated if assistance from others was required for recovery, resulted in hospitalization, or seizure. | ^[4]^ |
| Ghanim 2020 | <70 mg/dL | NR | NR |
| Brock 2019 | NR | NR | NR |

**Reference:**

[1] Seaquist ER, Anderson J, Childs B, Cryer P, Dagogo-Jack S, Fish L, Heller SR, Rodriguez H, Rosenzweig J, Vigersky R. Hypoglycemia and diabetes: a report of a workgroup of the American Diabetes Association and the Endocrine Society. *Diabetes Care* 2013, **36**(5)**:** 1384-1395.

[2] Draznin B, Aroda VR, Bakris G, Benson G, Brown FM, Freeman R, Green J, Huang E, Isaacs D, Kahan S, Leon J, Lyons SK, Peters AL, Prahalad P, Reusch JEB, Young-Hyman D. 6. Glycemic Targets: Standards of Medical Care in Diabetes-2022. *Diabetes Care* 2022, **45**(Suppl 1)**:** S83-s96.

[3] Association AD. 6. Glycemic Targets: Standards of Medical Care in Diabetes-2018. *Diabetes Care* 2018, **41**(Suppl 1)**:** S55-s64.

[4] Association AD. Professional Practice Committee: Standards of Medical Care in Diabetes-2019. *Diabetes Care* 2019, **42**(Suppl 1)**:** S3.
