# Supplementary material for: Glucagon-like peptide-1 receptor agonists as add-on therapy to insulin for type 1 diabetes mellitus
Source: Front Pharmacol. 2023 Mar 16;14:975880. doi: 10.3389/fphar.2023.975880 (PMC10797415; doi:10.3389/fphar.2023.975880)
Supplement: Supplementary file 1 [file DataSheet1.zip › Appendix 5. PRISMA flow diagram-1.docx]

Appendix 5. PRISMA flow diagram

45 full-text studies excluded

31 ineligible study design

3 short duration of follow-up

8 lack of primary endpoint

3 duplicates

Studies included in quantitative synthesis (meta-analysis)

(n=11)

## Eligibility

## Screening

## Identification

Records identified through database searching

(n=1379)

Pubmed: 86; Cochrane Central Register of Controlled Trials: 217; Embase: 1076

Records excluded following reading of title

and abstract because of failing to meet

inclusion criteria (n=1206)

## Included

Full-text articles assessed for eligibility (n=56)

Records after duplicates removed
(n=1262)

45 full-text studies excluded

31 ineligible study design 【^1-31^】

3 short duration of follow-up【^32-34^】

8 lack of primary endpoint【^35-42^】

3 duplicates【^43-45^】

11 studies included in quantitative synthesis (meta-analysis)【^46-56^】

Disagreements on eligibility of studies were settled by involving a third reviewer (Xiaochuan Wu), and these studies were highlighted.

**Reference：**

1. Ilkowitz, J.T., Katikaneni, R., Cantwell, M., Ramchandani, N., and Heptulla, R.A. (2016). Adjuvant Liraglutide and Insulin Versus Insulin Monotherapy in the Closed-Loop System in Type 1 Diabetes: A Randomized Open-Labeled Crossover Design Trial. Journal of diabetes science and technology 10, 1108-1114.

2. Pieber, T.R., Deller, S., Korsatko, S., Jensen, L., Christiansen, E., Madsen, J., and Heller, S.R. (2015). Counter-regulatory hormone responses to hypoglycaemia in people with type 1 diabetes after 4 weeks of treatment with liraglutide adjunct to insulin: a randomized, placebo-controlled, double-blind, crossover trial. Diabetes, obesity & metabolism 17, 742-750.

3. Pieber, T.R., Deller, S., Brunner, M., Jensen, L., Christiansen, E., Kiyomi, F., and Heller, S.R. (2013). Treatment with liraglutide as adjunct to insulin in type 1 diabetes; Effects on counter regulatory response to hypoglycaemia: A randomised, double blind, crossover trial. Diabetologia 56, S404-S405.

4. Heller, S.R., Korsatko, S., Gurban, J., Jensen, L., Christiansen, E., Kiyomi, F., and Pieber, T.R. (2013). Positive effects of liraglutide as adjunct to insulin in type 1 diabetes: Glycaemic control and safety in a randomised, double blind, placebo controlled crossover trial. Diabetologia 56, S7-S8.

5. Heller, S.R., Korsatko, S., Gurban, J., Jensen, L., Christiansen, E., Kiyomi, F., and Pieber, T.R. (2013). Liraglutide as adjunct to insulin in type 1 diabetes: Effects on glycemic control and safety in a randomized, double-blind, crossover trial. Diabetes 62, A258-A259.

6. Ballav, C., Dhere, A., Agbaje, O., Kennedy, I., Holman, R.R., and Owen, K.R. (2018). The effect of lixisenatide on post-prandial blood glucose and glucagon in type 1 diabetes. Diabetologia 61, S387.

7. Dube, M.C., D'Amours, M., and Weisnagel, S.J. (2016). Crossover, double-blind, unicentric, 52-week trial of liraglutide in type 1 diabetes. Diabetes 65, A76-A77.

8. Nct. (2007). Role of Exenatide in Type 1 Diabetes. <https://clinicaltrialsgov/show/NCT00456300>.

9. Nct. (2011). New Onset Type 1 Diabetes: role of Exenatide. <https://clinicaltrialsgov/show/NCT01269034>.

10. Nct. (2012). The Effect of Liraglutide Adjunct to Insulin on Glucagon Response to Hypoglycaemia in Subjects With Type 1 Diabetes. <https://clinicaltrialsgov/show/NCT01536665>.

11. Dubé, M.C., D'Amours, M., and Weisnagel, S.J. (2018). Beyond glycaemic control: A cross-over, double-blinded, 24-week intervention with liraglutide in type 1 diabetes. Diabetes, Obesity and Metabolism 20, 178-184.

12. Garg, M., Ghanim, H., Kuhadiya, N.D., Green, K., Hejna, J., Abuaysheh, S., Torre, B., Batra, M., Makdissi, A., Chaudhuri, A., et al. (2017). Liraglutide acutely suppresses glucagon, lipolysis and ketogenesis in type 1 diabetes. Diabetes Obes Metab 19, 1306-1311.

13. Sarkar, G., Alattar, M., Brown, R.J., Quon, M.J., Harlan, D.M., and Rother, K.I. (2014). Exenatide treatment for 6 months improves insulin sensitivity in adults with type 1 diabetes. Diabetes care 37, 666‐670.

14. Dube, M.C., D'Amours, M., and Weisnagel, S.J. (2018). Beyond glycaemic control: A cross-over, double-blinded, 24-week intervention with liraglutide in type 1 diabetes. Diabetes Obes Metab 20, 178-184.

15. Pozzilli, P., Leslie, R.D., Peters, A.L., Buzzetti, R., Shankar, S.S., Milicevic, Z., Pavo, I., Lebrec, J., Martin, S., and Schloot, N.C. (2018). Dulaglutide treatment results in effective glycaemic control in latent autoimmune diabetes in adults (LADA): A post-hoc analysis of the AWARD-2, -4 and -5 Trials. Diabetes, obesity & metabolism 20, 1490-1498.

16. Kielgast, U., Holst, J.J., and Madsbad, S. (2010). Treatment of type 1 diabetic patients with residual beta cell function with the once-daily glucagon-like peptide-1 analogue liraglutide. Diabetologia 53, S340.

17. Sarkar, G., Alattar, M., Brown, R.J., Quon, M.J., Harlan, D.M., and Rother, K.I. (2012). Exenatide treatment for 6 months improves insulin sensitivity in adults with type 1 diabetes mellitus. Diabetes 61, A17.

18. Curtis, L., Holt, H., Richardson, T., Knott, J., and Partridge, H. (2016). GLP-1 analogue use in patients with sub-optimally controlled type 1 diabetes or obesity improves weight and HbA1c. Practical Diabetes 33, 13-17.

19. Almazrouei, R., Alkaabi, F., Lessan, N., and Barakat, M.T. (2017). Glucagon like peptide-1 agonist treatment in patients with Type 1 diabetes: effects on weight and glycaemic control in an Emirati population. Diabetic medicine 34, 186‐187.

20. Behme, M.T., Dupré, J., and McDonald, T.J. (2003). Glucagon-like peptide I improved glycemic control in type I diabetes. BMC endocrine disorders 3.

21. Shah, M.K., Danjuma, M., Saeed, T., and Khan, E.G. (2014). An investigation into the efficacy of glucagon-like peptide 1 (GLP-1) analogues in obese patients with Type 1 diabetes. Diabetic Medicine 31, 178.

22. Varanasi, A., Bellini, N., Rawal, D., Vora, M., Makdissi, A., Dhindsa, S., Chaudhuri, A., and Dandona, P. (2011). Liraglutide as additional treatment for type 1 diabetes. European Journal of Endocrinology 165, 77-84.

23. Kuhadiya, N., Malik, R., Bellini, N., Patterson, J., Traina, A., Makdissi, A., and Dandona, P. (2013). Liraglutide as additional treatment to insulin in obese patients with type 1 diabetes mellitus. Endocrine Practice 19, 963-967.

24. Traina, A.N., Lull, M.E., Hui, A.C., Zahorian, T.M., and Lyons-Patterson, J. (2014). Once-weekly exenatide as adjunct treatment of type 1 diabetes mellitus in patients receiving continuous subcutaneous insulin infusion therapy. Canadian Journal of Diabetes 38, 269-272.

25. Marie, C., D'Amours, M., and Weisnagel, S.J. (2014). Crossover, double-blind, 52 week trial of liraglutide in type 1 diabetes. Canadian Journal of Diabetes 38, S56.

26. Singh, B.M., Dudley, J., and Gillani, S.R. (2013). An audit of glucagon-like peptide 1 (GLP-1) agonist use in obese people with Type 1 diabetes. Diabetic Medicine 30, 81.

27. Kuhadiya, N.D., Prohaska, B., Ghanim, H., and Dandona, P. (2018). Addition of GLP-1 therapy to insulin in c-peptide-positive patients with type 1 diabetes. Diabetes 67, LB31.

28. Dupre, J., Behme, M.T., and McDonald, T.J. (2004). Exendin-4 normalized postcibal glycemic excursions in type 1 diabetes. Journal of clinical endocrinology and metabolism 89, 3469‐3473.

29. Nct. (2013). Effect of Liraglutide on Automated Closed-loop Glucose Control in Type 1 Diabetes. <https://clinicaltrialsgov/show/NCT01856790>.

30. Nct. (2019). Effect of GLP-1 on Microvascular Insulin Responses in Type 1 Diabetes. <https://clinicaltrialsgov/show/NCT04133922>.

31. Frandsen, C.S., Ostergaard, L., Dejgaard, T.F., Sondergaard, B., Wewer Albrechtsen, N.J., Holst, J.J., and Madsbad, S. (2016). The effect of glucagon-like peptide-1 receptor agonists on postprandial glucagon secretion independent of the gastric emptying rate. Diabetologia 59, S381‐.

32. Kielgast, U., Krarup, T., Holst, J.J., and Madsbad, S. (2011). Four weeks of treatment with liraglutide reduces insulin dose without loss of glycemic control in type 1 diabetic patients with and without residual β-cell function. Diabetes Care 34, 1463-1468.

33. Ceriello, A., Novials, A., Ortega, E., Canivell, S., La Sala, L., Pujadas, G., Esposito, K., Giugliano, D., and Genovese, S. (2013). Glucagon-like peptide 1 reduces endothelial dysfunction, inflammation, and oxidative stress induced by both hyperglycemia and hypoglycemia in type 1 diabetes. Diabetes care 36, 2346‐2350.

34. Hari Kumar, K.V.S., Shaikh, A., and Prusty, P. (2013). Addition of exenatide or sitagliptin to insulin in new onset type 1 diabetes: A randomized, open label study. Diabetes Research and Clinical Practice 100, e55-e58.

35. Dejgaard, T.F., Frandsen, C.S., Kielgast, U., Andersen, H.U., Thorsteinsson, B., Krarup, T., Holst, J.J., and Madsbad, S. (2019). Liraglutide preserved insulin secretion in adults with newly diagnosed type 1 diabetes: the newlira trial. Diabetes 68.

36. Nct. (2015). Liraglutide Effect on Beta-cell Function in C-peptide Positive Type 1 Diabetes. <https://clinicaltrialsgov/show/NCT02617654>.

37. Nct. (2017). Liraglutide as an Additional Treatment to Insulin in Patients With Autoimmune Diabetes Mellitus. <https://clinicaltrialsgov/show/NCT03011008>.

38. Nct. (2016). Incretin-based Therapy in Early Diagnosed Type 1 Diabetes. <https://clinicaltrialsgov/show/NCT02908087>.

39. Jiang, L.L., Wang, S.Q., Ding, B., Zhu, J., Jing, T., Ye, L., Lee, K.O., Wu, J.D., and Ma, J.H. (2018). The effects of add-on exenatide to insulin on glycemic variability and hypoglycemia in patients with type 1 diabetes mellitus. Journal of endocrinological investigation 41, 539-547.

40. Nct. (2009). Effect of GLP-1 on Insulin-dose, Risk of Hypoglycemia and Gastric Emptying Rate in Patients With Type 1 Diabetes. <https://clinicaltrialsgov/show/NCT00993720>.

41. Nct. (2008). Effect of GLP-1 and GIP on Insulin Secretion in Type-1 Diabetes Mellitus. <https://clinicaltrialsgov/show/NCT00603031>.

42. Nct. (2018). Dulaglutide and Insulin MicrosecretiON in Type 1 Diabetes. <https://clinicaltrialsgov/show/NCT03668470>.

43. Dejgaard, T.F., Johansen, N.B., Frandsen, C.S., Asmar, A., Tarnow, L., Knop, F.K., Madsbad, S., and Andersen, H.U. (2016). Cardiovascular effects of liraglutide in patients with type 1 diabetes: A randomised, double-blinded placebo-controlled trial (Lira-1). Diabetologia 59, S356-S357.

44. Dejgaard, T.F., Knop, F.K., Tarnow, L., Frandsen, C.S., Hansen, T.S., Almdal, T., Holst, J.J., Madsbad, S., and Andersen, H.U. (2015). Efficacy and safety of the glucagon-like peptide-1 receptor agonist liraglutide added to insulin therapy in poorly regulated patients with type 1 diabetes - A protocol for a randomised, double-blind, placebo-controlled study: The Lira-1 study. BMJ Open 5.

45. Dejgaard, T.F., Knop, F.K., Tarnow, L., Frandsen, C.S., Hansen, T.S., Almdal, T., Holst, J.J., Madsbad, S., and Andersen, H.U. (2015). Efficacy and safety of the glucagon-like peptide-1 receptor agonist liraglutide added to insulin therapy in poorly regulated patients with type 1 diabetes--a protocol for a randomised, double-blind, placebo-controlled study: the Lira-1 study. BMJ open 5, e007791.

46. <1.Addition of liraglutide to insulin in patients with type 1 diabetes_ a randomized placebo-controlled clinical trial of 12 weeks.pdf>.

47. <2.Efficacy and safety of liraglutide added to insulin treatment in type 1 diabetes_ the ADJUNCT ONE treat-to-target randomized trial.pdf>.

48. <3.Efficacy and safety of liraglutide added to capped insulin treatment in subjects with type 1 diabetes_ the ADJUNCT TWO randomized trial.pdf>.

49. <8.Twelve-week treatment with liraglutide as add-on to insulin in normal-weight patients with poorly controlled type 1 diabetes_ a randomized, placebo-controlled, double ….pdf>.

50. Brock, C., Hansen, C.S., Karmisholt, J., Moller, H.J., Juhl, A., Farmer, A.D., Drewes, A.M., Riahi, S., Lervang, H.H., Jakobsen, P.E., et al. (2019). Liraglutide treatment reduced interleukin 6 in adults with type 1 diabetes but did not improve established autonomic or polyneuropathy. British journal of clinical pharmacology.

51. Dejgaard, T.F., Frandsen, C.S., Hansen, T.S., Almdal, T., Urhammer, S., Pedersen-Bjergaard, U., Jensen, T., Jensen, A.K., Holst, J.J., Tarnow, L., et al. (2016). Efficacy and safety of liraglutide for overweight adult patients with type 1 diabetes and insufficient glycaemic control (Lira-1): a randomised, double-blind, placebo-controlled trial. The lancet Diabetes & endocrinology 4, 221‐232.

52. Dejgaard, T.F., Schmidt, S., Frandsen, C.S., Vistisen, D., Madsbad, S., Andersen, H.U., and Norgaard, K. (2019). Liraglutide reduces hyperglycaemia and body weight in overweight, dysregulated insulin-pump-treated patients with type 1 diabetes: the Lira Pump trial - a randomized, double-blinded, placebo-controlled trial. Diabetes, obesity & metabolism.

53. Ghanim, H., Batra, M., Green, K., Abuaysheh, S., Hejna, J., Makdissi, A., Borowski, R., Kuhadiya, N.D., Chaudhuri, A., and Dandona, P. (2020). Liraglutide treatment in overweight and obese patients with type 1 diabetes: A 26-week randomized controlled trial; mechanisms of weight loss. Diabetes Obes Metab 22, 1742-1752.

54. Herold, K.C., Reynolds, J., Dziura, J., Baidal, D., Gaglia, J., Gitelman, S.E., Gottlieb, P.A., Marks, J., Philipson, L.H., Pop-Busui, R., et al. (2020). Exenatide extended release in patients with type 1 diabetes with and without residual insulin production. Diabetes Obes Metab 22, 2045-2054.

55. Johansen, N.J., Dejgaard, T.F., Lund, A., Schluntz, C., Frandsen, C.S., Forman, J.L., Wewer Albrechtsen, N.J., Holst, J.J., Pedersen-Bjergaard, U., Madsbad, S., et al. (2020). Efficacy and safety of meal-time administration of short-acting exenatide for glycaemic control in type 1 diabetes (MAG1C): a randomised, double-blind, placebo-controlled trial. Lancet Diabetes Endocrinol 8, 313-324.

56. Pozzilli, P., Bosi, E., Cirkel, D., Harris, J., Leech, N., Tinahones, F.J., Vantyghem, M.C., Vlasakakis, G., Ziegler, A.G., and Janmohamed, S. (2020). Randomized 52-week Phase 2 Trial of Albiglutide Versus Placebo in Adult Patients With Newly Diagnosed Type 1 Diabetes. The Journal of clinical endocrinology and metabolism 105.
