# Supplementary material for: Glucagon-like peptide-1 receptor agonists as add-on therapy to insulin for type 1 diabetes mellitus
Source: Front Pharmacol. 2023 Mar 16;14:975880. doi: 10.3389/fphar.2023.975880 (PMC10797415; doi:10.3389/fphar.2023.975880)
Supplement: Supplementary file 1 [file DataSheet1.zip › Appendix 6. Risk of bias.docx]

Appendix 6. Risk of bias

| Study | Random Sequence Generation | Allocation Concealment | Blinding | Incomplete Outcome Data | Selective Reporting | Other bias |
| --- | --- | --- | --- | --- | --- | --- |
| Kuhadiya 2016 | H | L | L | H | U | H |
| Mathieu 2016 | L | L | L | U | L | L |
| Ahren 2016 | L | L | L | U | L | U |
| Dejgaard 2015 | L | L | L | L | L | L |
| Johansen 2020 | L | L | L | L | L | L |
| Dejgaard 2020 | L | L | L | L | L | L |
| Pozzilli 2020 | L | L | L | L | L | H |
| Frandsen 2015 | L | L | L | U | L | L |
| Herold 2020 | L | L | L | U | U | L |
| Ghanim 2020 | L | L | L | H | H | H |
| Brock 2019 | L | L | L | H | H | H |

L = Low Risk; H = High Risk; U = Unclear Risk.
