# Supplementary material for: Glucagon-like peptide-1 receptor agonists as add-on therapy to insulin for type 1 diabetes mellitus
Source: Front Pharmacol. 2023 Mar 16;14:975880. doi: 10.3389/fphar.2023.975880 (PMC10797415; doi:10.3389/fphar.2023.975880)
Supplement: Supplementary file 1 [file DataSheet1.zip › Appendix 1. Comparator table of all agents.docx]

**Appendix 1. Comparator table of all agents**

| **Compound** | **Structure Basis** | **Half-life** | **Size** | **Pharmacokinetic Classification** | **Administration** |
| --- | --- | --- | --- | --- | --- |
| Exenatide twice daily | Exendin 4 | 2.4hrs | 4.2kDa | Short-acting GLP-1 RAs | Twice daily |
| Liraglutide | GLP1 | 13 hrs | 3.8KDa | Long-acting GLP-1 RAs | Once daily |
| Exenatide once weekly | Exendin 4 | NA | NA | Long-acting GLP-1 RAs | Once weekly |
| Albiglutide | GLP1 | 5 days | 73.0KDa | Long-acting GLP-1 RAs | Once weekly |
